# Artificial intelligence and peripheral neuropathies: Strategies for the development, application, and repair of regenerative biomaterials

**DOI:** 10.4103/NRR.NRR-D-25-00561

**Published:** 2025-09-29

**Authors:** Zixu Zhang, Yi Yao, Zitao Wang, Huiyuan Bai, Maorong Jiang, Min Cai, Dengbing Yao

**Affiliations:** 1 School of Life Sciences, Key Laboratory of Neuroregeneration of Jiangsu and Ministry of Education, Co-innovation Center of Neuroregeneration, Nantong University, Nantong, Jiangsu Province, China; 2 School of Public Health, Nantong University, Nantong, Jiangsu Province, China; 3 Medical School of Nantong University, Nantong, Jiangsu Province, China

**Keywords:** 3D printing, artificial intelligence, biomaterials, bioresorbable scaffolds, deep learning, machine learning, nerve conduits, nerve regeneration, peripheral nerve injuries, tissue engineering

## Abstract

Traditional repair methods for peripheral neuropathies, such as autologous and allogeneic nerve grafts, face limitations, while peripheral nerve regeneration materials have emerged as a promising alternative. However, current biomaterials are mostly single-functional and insufficient in modulating the regenerative microenvironment. This review explores the application of artificial intelligence in the development of neural regenerative biomaterials, focusing on material design, performance prediction, and virtual experiments. Artificial intelligence has the potential to optimize material properties through machine learning and deep learning, predict material performance, and enhance nerve regeneration. Recent studies have demonstrated the ability of artificial intelligence to design biomaterials with improved biocompatibility and mechanical properties, as well as to accurately predict outcomes of nerve regeneration. However, several challenges remain, such as data integration, algorithm complexity, and ensuring clinical translation. The promising future of intelligent research and development in biomaterials lies in personalized treatment strategies, coupled with the integration of advanced technologies such as artificial intelligence and 3D bioprinting, to create more efficient neural repair materials. This review highlights the transformative potential of artificial intelligence in advancing peripheral nerve repair and improving patient outcomes.

**Facts**

• Machine learning can drive high-throughput screening and optimization of 3D printing parameters.

• Multimodal artificial intelligence models integrate genomic, imaging, and biomechanical data to enable personalized nerve conduit customization for patient-specific nerve defects. Artificial intelligence can complete material–structure–performance iterations in hours that traditionally take weeks, considerably improving the efficiency of nerve conduit design.

• An artificial intelligence–based virtual cell simulation platform can predict material–host interactions before implantation, reduce the use of animal experiments, and accelerate the translation from the laboratory to the clinic.

**Open questions**

• How can we establish standardized data interfaces across centers and devices to mitigate batch effects and confounding from clinical heterogeneity caused by differences in sample sources during artificial intelligence training?

• When the degradation rate of an artificial intelligence–designed biodegradable nerve conduit does not match the patient’s actual nerve regeneration rate, how should a real-time closed-loop feedback mechanism be constructed to dynamically adjust the material properties?

• In artificial intelligence–assisted personalized nerve repair, how can the “black-box” nature of model decisions be reconciled with medical explainability requirements to meet the transparency standards of regulatory and ethical reviews across different countries?

## Introduction

Peripheral neuropathies are common clinical conditions that can arise from trauma, surgery, or disease (Yao et al., 2023a). They can cause motor dysfunction, paresthesia, and autonomic dysfunction, severely affecting patients’ daily lives. Peripheral nerve injury (PNI) is a prototypical peripheral neuropathy (Ding et al., 2024; Lv et al., 2024). Pathological changes include impaired axoplasmic transport, axonal degeneration, Schwann cell (SC) injury, segmental demyelination, and complete Wallerian degeneration. Peripheral nerve repair (PNR) remains a major clinical challenge (Liu et al., 2019a, b). It is a complex biological process involving cell proliferation, differentiation, and migration; axonal regeneration; myelin remodeling; and interactions among multiple cell types and signaling pathways (Chen et al., 2024a, b). Despite advances in elucidating its molecular mechanisms, they are not fully understood, and significant bottlenecks persist in clinical practice (Xue et al., 2021). In recent years, the rapid development of artificial intelligence (AI) has opened new possibilities for addressing these challenges (Guo et al., 2024). The use of AI in materials science, biomedical engineering, and clinical medicine has become a research focus, offering new strategies for the development, application, and optimization of biomaterials for peripheral nerve regeneration (Zampieri et al., 2019; Alborghetti et al., 2024). This review explores the application of AI in peripheral nerve diseases, with a focus on its strategies for developing, applying, and advancing regenerative biomaterials.

Advances have been made in PNR research, yet numerous challenges remain. For patients with severe nerve injuries and large nerve gaps, nerve grafts are essential to bridge the defects (Yu et al., 2024a, b). However, autologous nerve transplantation faces several challenges, including limited donor nerve availability, high donor-site morbidity, size mismatch between donor and recipient nerves, and partial nerve dysfunction (Gu et al., 2011). Allogeneic nerve transplantation, while alleviating the shortage of donor nerves to some extent, necessitates immunosuppression and is associated with immune rejection and ethical concerns. Although evidence exists that adding host SCs to allogeneic nerve grafts can prevent immune rejection (Ray and Mackinnon, 2010), the clinical feasibility and specific implementation of this protocol require further study. PNR materials (PNRMs) have shown potential in promoting nerve regeneration by enhancing stem and neural cell proliferation, improving neuronal migration, reducing inflammation, and guiding axonal regeneration (MankavI et al., 2023). However, most existing PNRMs have limited functionality and struggle to meet multiple demands simultaneously. Many biomaterials do not adequately mimic the natural nerve tissue microenvironment and therefore fail to provide optimal conditions for regeneration. Moreover, the development of PNRMs is complicated by challenges in material selection, design, manufacturing processes, performance optimization, and clinical translation. Beyond material functionality, the cost of design and the need for personalized treatment have also become prominent concerns. There is an urgent need to develop more effective, safer, and more personalized repair methods to improve patients’ prognosis and quality of life. The emergence of AI technologies offers new possibilities for meeting these challenges.

AI technologies hold broad promise for developing biomaterials for PNR. Using machine learning (ML) and deep learning (DL) algorithms, AI can analyze large volumes of materials-property data to predict clinical performance and thereby optimize material selection and design (Isayev et al., 2017; Zhang et al., 2024a, b). For example, AI can model complex relationships among factors in the regeneration process—such as the extracellular matrix (ECM), neurotrophic factors (NTFs), and inflammatory responses—to build multifactor-coupled models that accurately predict regeneration outcomes under different conditions (Omigbodun et al., 2024). Additionally, AI can optimize manufacturing parameters in 3D bioprinting to improve implantation accuracy and enhance the efficiency of tissue regeneration (Huang et al., 2021). In clinical applications, AI can integrate patients’ genomic data, imaging results, and clinical symptoms to provide personalized treatment plans and improve therapeutic outcomes (Zampieri et al., 2019). Despite this potential, significant challenges remain for clinical translation. Ethically, AI applications raise concerns about privacy and data security. Technically, model complexity and high data requirements can complicate development and implementation (Omigbodun et al., 2024). In terms of cost, developing and deploying AI technologies requires substantial financial investment, which may limit widespread adoption in some regions (Huang et al., 2021). Therefore, future research should seek a balance among technological optimization, ethical governance, and cost control to promote the sustainable development of AI technologies in PNR.

Future research should focus on overcoming bottlenecks in the application of AI technologies in PNR. On one hand, AI models need further optimization to improve their analytical capabilities and predictive accuracy for complex biomedical datasets (Isayev et al., 2017; Zhang et al., 2024a, b). On the other hand, multidisciplinary collaboration should be strengthened by integrating materials science, biomedical engineering, and clinical medicine to develop more efficient and safer regenerative biomaterials (Huang et al., 2021). In addition, ethical guidelines and policies should be established to ensure the responsible and safe clinical use of AI technologies.

This review focuses on AI strategies for addressing peripheral nerve diseases through the development, application, and repair of regenerative biomaterials. By optimizing material selection, design, and microenvironmental regulation, AI offers novel approaches for tackling the complexities of PNR. Although these technologies hold great potential, they still face numerous challenges in clinical application. The potential of AI to enhance therapeutic outcomes and improve patient prognosis should not be underestimated. Future research must balance technological optimization, ethical oversight, and cost control to promote the sustainable development of AI in the field of PNR.

## Search Strategy

The Web of Science was selected as the primary database. We searched for literature on the application of AI in peripheral nerve disease research from January 1, 2000, to May 2, 2025. The subject search method was used to capture titles, abstracts, and keywords. The specific search formulas are provided in **Additional Table 1**. This approach aimed to offer a comprehensive overview of current research, identify gaps, and explore future directions. We established criteria to filter the literature. Eligible studies involved AI technologies, peripheral nerve diseases (e.g., PNI and nerve regeneration), and nerve regeneration materials; were published in English between January 1, 2000, and May 2, 2025; and were research or review articles. Studies published outside this period were excluded. After screening titles, abstracts, and full texts against these criteria, we identified the final set of relevant literature.

**Additional Table 1:** Literature retrieval strategy

Steps	Search formula
**#1**	TS= ("Peripheral Nervous System Diseases" OR "Peripheral Neuropathies" OR "Neuroregeneration" OR "Nerve Repair" OR "Neuroprotection")
**#2**	TS= ("Artificial intelligence" OR "Machine Learning" OR "Deep Learning" OR "Natural Language Processing" OR "Robotics" OR "Neural Networks")
**#3**	TS= ("Neural Regeneration Materials" OR "Nerve Guidance Conduits" OR "Biomaterials" OR "Tissue Engineering" OR "Neurotrophic Factors" OR "Carbon Nanomaterials" OR "Electrical Stimulation" OR "Physical Modulation"OR "Scaffolds" OR "Biodegradable" OR "Nanotechnology" OR "Biomimetic" OR "Neuroprosthetics" OR "Neurotissue Engineering")
**#4**	TS= ("Neurotissue Engineering" OR "Neuroprosthetics" OR "Neurotrophic Factors" OR "Nerve Regeneration Scaffolds" OR "Biodegradable Polymers" OR "Conductive Biomaterials" OR "Nanotechnology for Neural Repair" OR "Biomimetic Scaffolds" OR "Neuronal Interface" OR "Neuronal Signaling" OR "Neuronal Differentiation" OR "Neuronal Survival" OR "Neuronal Integration")
**#5**	#1 AND #2
**#6**	#2 AND #3
**#7**	#2 AND #4

## Biomaterials for Peripheral Nerve Regeneration

### Overview of peripheral nerve repair materials

At present, the main treatment methods for PNI include autologous nerve transplantation, allograft nerve transplantation, and implantation of artificial repair materials. However, these approaches have limitations. For example, the limited availability of donor nerves, donor–recipient mismatch, tissue adhesion, functional disorders after transplantation, the risk of secondary surgery, and the potential to damage healthy nerves and cause additional surgical wounds all contribute to suboptimal therapeutic outcomes (Chang et al., 2017). Allogeneic nerve transplantation, as an alternative to autologous transplantation, offers advantages such as preserving autologous nerves and avoiding secondary surgery. However, immune rejection and ethical concerns limit its widespread use. Currently, biomaterials have been widely studied as an alternative strategy to repair peripheral nerve defects. By selecting appropriate biomaterials, constructing nerve conduits, and promoting nerve and axonal regeneration through strategies such as morphological modification of the conduit lumen, incorporation of growth factors, and improvement of the regenerative microenvironment, repair outcomes can be enhanced. In a study of Sprague–Dawley rats with 1 cm nerve defects, researchers investigated vascularized nerve conduits for peripheral nerve repair. Eight weeks after surgery, the vascularized conduits were more effective in promoting nerve regeneration than autologous nerve grafts (Yapici et al., 2017).

These biomaterials are mainly divided into nondegradable and degradable materials. Nondegradable materials are less studied because they require secondary surgery for removal and may cause friction with surrounding tissues. Biodegradable materials are further classified as natural or synthetic. Natural materials mainly include decellularized matrix, collagen, and chitosan, while synthetic materials include polymers such as polyurethane, polylactic acid (PLA), and polycaprolactone (Zou et al., 2024). Incorporating stem cells, NTFs, ECM, proteins, and conductive materials into these nerve grafts can provide physical support, regulate the regenerative microenvironment, and deliver biological signals for nerve regeneration, as well as electrical and optogenetic stimulation (Takaoka et al., 2022). For instance, ECM plays a crucial role in neural conduits by regulating cell growth, migration, differentiation, and secretion, and by promoting tissue repair and regeneration (Gattazzo et al., 2014). In addition to providing physical cues, the neural ECM contains proteins such as laminin and fibronectin that influence cell migration and differentiation (Sheppard et al., 1991). For example, fibronectin promotes cell adhesion and growth (Matesz et al., 2005), while laminin stimulates axonal regeneration and activates SCs (Gu et al., 2011). Thus, these approaches provide a stable scaffold for axonal growth, enable precise regulation of neural activity, and create a microenvironment conducive to regeneration, thereby promoting axonal regrowth, reconnection with target neurons, and ultimately restoration of peripheral nerve structure and function (Gu et al., 2014).

### Mechanism of biomaterials in peripheral nerve regeneration

#### Promotion of nerve fiber regeneration

Biomaterials can mimic the structure of neural pathways by forming longitudinal alignments. These alignments act as three-dimensional scaffolds that provide pathways for axonal growth and guide the extension of regenerating axons, thereby promoting cell migration and the formation of neural connections. For example, polyurethane conduits—owing to their excellent biocompatibility, tunable mechanical properties, and ability to support neural regeneration through functional modification (Wang et al., 2022)—can bridge nerve defects and provide the necessary environment for neural regeneration (Lan et al., 2025). Gu et al. (2021) adjusted the groove pattern on silk fibroin gel and found that a groove size of 30 μm effectively enhanced axonal elongation and achieved optimal nerve regeneration.

Concurrently, biomaterials can carry and release NTFs, such as nerve growth factor (NGF), brain-derived neurotrophic factor (BDNF), neurotrophin-3 (NT-3), and glial cell line-derived neurotrophic factor (GDNF). These factors protect damaged nerve cells and, in combination with stem cells, can effectively promote neuronal differentiation, synaptic growth, and synaptic connections (Rafieva and Gasanov, 2016). For example, after spinal cord root avulsion injury, increasing GDNF levels can significantly promote motor neuron survival, stimulate distal axonal growth, and enhance reinnervation of target muscles and recovery of autonomous function (Eggers et al., 2019). A recent study suggests that NTFs can be delivered by coupling them to neural repair conduits or by stimulating neurons within the conduit lumen (Zhang et al., 2000). As an essential trophic factor for nervous system development, BDNF supports neuronal survival during naturally occurring cell death; without BDNF, neurons have difficulty regenerating. BDNF can enhance the proliferative activity of neuronal precursor cells, promote neuronal migration to the injury site, and exert antiapoptotic effects (Cacialli et al., 2018). Previous studies have shown that BDNF-loaded composite catheters can maintain BDNF bioactivity for at least 3 months and promote regeneration across 10-mm sciatic nerve defects in rats (Su et al., 2020; Yang et al., 2020). Long et al. (2021) cross-linked heparin with collagen tubes to construct a nerve-guidance catheter for NGF delivery; 12 weeks after surgery, the catheter significantly promoted axonal growth and SC proliferation. Hydrogel materials have been used as carriers to deliver NTFs into peripheral nerve defects, filling the injured area and providing a suitable microenvironment for regeneration. For instance, Lu et al. (2019) prepared nanofiber hydrogel conduits loaded with growth factors such as NGF by inducing filamentous protein nanofibers with an electric field. Immunofluorescence showed that this hydrogel nerve conduit optimized the microenvironment for nerve regeneration. The addition of these growth factors enhanced conduit performance, greatly promoted the proliferation and differentiation of pluripotent stem cells, and accelerated axon formation (Wu et al., 2016).

In addition, biomaterials can provide electrical stimulation by integrating electrodes, thereby promoting axonal elongation and nerve regeneration (Meyer et al., 2016). The combination of electrical stimulation and biomaterials can significantly improve motor, electrophysiological, and morphological recovery in damaged nerves (Wang et al., 2020). For example, a nerve conduction catheter made of hydroxyethyl cellulose, soy protein isolate, and polyaniline sponge, prepared by Liu et al. (2021) and combined with electrical stimulation, effectively promoted nerve repair. Park et al. (2020) prepared a conductive nerve catheter using graphene oxide combined with methacrylated gelatin, and the results showed a strong promoting effect on PNI regeneration.

Given the complexity of nerve regeneration mechanisms, AI can remarkably enhance the design and application of biomaterials. Specifically, AI algorithms can optimize structural arrangements to promote axonal growth and myelination and fine-tune the release of NTFs for sustained, controlled delivery (Catrina et al., 2013; Gu et al., 2021).

#### Regulation of cellular signaling pathways

During PNR, cell signaling pathways play a crucial role. Among them, the PI3K/Akt pathway is considered a key transduction system that regulates the immune microenvironment, inhibits apoptosis, promotes the proliferation and differentiation of nerve cells, and supports angiogenesis and axonal growth (Kemp et al., 2018). Wang et al. (2024) found that electroactive neural conduits can modulate the immune microenvironment by activating the PI3K/Akt–Nrf2 pathway, thereby promoting macrophage polarization toward an anti-inflammatory M2 phenotype and enhancing macrophage paracrine signaling to promote SC recruitment and myelination. Meanwhile, Zhang et al. (2014a, b) developed a Li–Ca–Si bioceramic-based bioink that releases multiple active ions and activates the PI3K/Akt pathway, thereby promoting the differentiation of neural stem cells into neurons and inducing neuronal maturation at the morphological, molecular, and electrophysiological levels (Zhang et al., 2024a, b). In addition, the Wnt/β-catenin pathway also plays an important role in PNR. Chai et al. (2022) applied a self-developed multilevel aligned fibrin nanofiber hydrogel to the spinal cord and PNI, which provided directional structural and biochemical cues that activated the Wnt/β-catenin pathway and promoted nerve regeneration. Previous studies have also shown that chitosan/silk fibroin scaffolds enhance the expression of genes related to nerve regeneration, such as activated transcription factor 3, cyclin-dependent kinase inhibitor 1A, interleukin-6, and insulin-like growth factor 1 (Jiang et al., 2023; Xue et al., 2024). Activation of these genes can enhance SC migration and survival and inhibit apoptosis of damaged SCs via the Bcl-2/Bax/caspase-3 signaling pathway, thereby exerting neuroprotective effects. Additionally, noncanonical feedback loops involving necroptosis-related proteins such as RIP3 and MLKL have been identified as critical in neuronal necroptosis (Wang et al., 2025b), suggesting that biomaterials designed to modulate these pathways could enhance nerve regeneration by reducing cell death.

AI can help model and predict how biomaterials affect signaling pathways by analyzing cellular response data, thereby optimizing material design for better therapeutic outcomes. For example, it can simulate the effect of bioactive ions on nerve regeneration, as shown in a recent study (Wu et al., 2024).

### Design requirements for biomaterials

Due to the interactions among multiple signaling pathways and cell types involved in PNR, the design requirements for related biomaterials are more stringent. Biomaterials used to fill or guide damaged tissue repair must be biocompatible. They should be highly compatible with nerve tissue, not trigger immune rejection or inflammation, and maintain a stable microenvironment for nerve regeneration (Liu et al., 2023). A stable microenvironment can enhance mitochondrial function, increase ATP synthesis, and alleviate oxidative stress, thereby supporting high-energy-demand processes (Babetto et al., 2020). In addition, it can regulate ion channels and bioelectrical activity, aiding the restoration of neural signaling. This is crucial for restoring neuronal function and promoting neural repair.

Biomaterials also need appropriate mechanical properties. First, they must provide sufficient mechanical strength. During repair, the material must support the surrounding nerve tissue to prevent deformation or damage under external forces and ensure that regeneration channels remain unobstructed (Patel et al., 2018). Second, because peripheral nerves undergo stretching and bending during movement, biomaterials should have good flexibility and elasticity to accommodate the dynamics of injured nerves. Moreover, Zhang et al. (2018) indicated that material mechanics can influence cell migration and regeneration. For example, polydimethylsiloxane has excellent biocompatibility and mechanical properties and can effectively promote nerve growth and cell migration (Li et al., 2014).

Material structure and morphology—such as permeability, porosity, degradation rate, and the optimal wall thickness of nerve conduits—can affect repair outcomes (Kim et al., 2020). Porous structures increase specific surface area, provide space for neural tissue regeneration, and offer more attachment sites for nerve cell growth. They also facilitate the exchange of nutrients and metabolites, prevent ingrowth of surrounding tissues, and promote nerve regeneration (Zhao et al., 2017). For example, silk–magnesium composite braided porous nerve conduits support nerve fiber growth and angiogenesis (Zhang et al., 2021a, b). Incorporating directional features, such as microgrooves, can guide nerve fibers to grow along predetermined paths, improving the order and efficiency of regeneration and reducing misdirected regeneration (Zhang et al., 2022). Additionally, studies have shown that multichannel conduits for peripheral nerve repair can improve axonal elongation, thereby enhancing tissue regeneration and effectively promoting nerve repair (Dinis et al., 2015; Carvalho et al., 2019). However, their performance still lags behind autologous nerve repair. Therefore, conduit designs should be further refined with more biomimetic features to improve outcomes. In addition, the ideal biomaterial should be appropriately biodegradable: it should provide necessary support and protection in the early stages of regeneration and then gradually degrade and be absorbed as the nerve repairs and matures, avoiding complications from long-term implantation (Li et al., 2014). For example, poly(L-lactide-co-ε-caprolactone) is biodegradable; while supporting nerve regeneration, it gradually degrades. Its hollow tubular structure can bridge nerve defects and provide guidance and support for axonal growth (Hibbitts et al., 2022).

**Figure 1:**
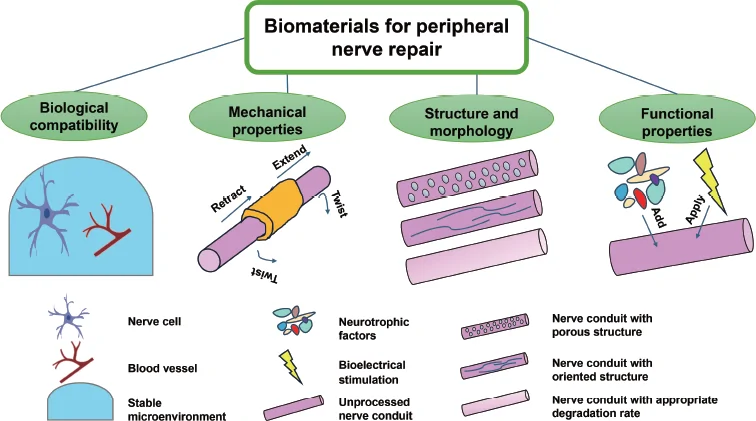
Mechanism of biomaterials in peripheral nerve regeneration. The diagram categorizes the design requirements of biomaterials into four main categories: (1) Biological compatibility, emphasizing the provision of a stable microenvironment for nerve cells; (2) Mechanical properties, including flexibility, mechanical strength, and adaptability of the material; (3) Structure and morphology, requiring the material to have a porous structure, oriented structure, and an appropriate degradation rate; (4) Functional properties, highlighting the role of bioelectrical stimulation and neurotrophic factors. These criteria collectively ensure that the biomaterials can effectively support the repair and regeneration of peripheral nerves.

Biomaterials should also offer functional capabilities, such as loading NGF, BDNF, NT-3, GDNF, and other NTFs to provide continuous trophic support, promote neuronal differentiation, and stimulate synaptic growth. For example, Li et al. (2020) showed that exogenous NGF activates autophagy in dedifferentiated SCs, accelerates myelin debris clearance and phagocytosis, and promotes early axonal and myelin regeneration after PNI. Requirements for growth factors vary across stages of nerve regeneration, so the types and concentrations should be dynamically adjusted. Different NTFs can act synergistically to regulate regeneration, optimizing the microenvironment at each stage and improving repair outcomes (Madduri et al., 2010). Catrina et al. (2013) investigated the release kinetics of GDNF and NGF from silk–collagen scaffolds and, by adjusting the silk-to-collagen ratio, achieved independent control over the release of each factor, optimizing the microenvironment at different stages, and ultimately improving tissue repair. In addition to NTFs, various drugs—such as urolithin A, FK506, curcumin, retinoic acid, dexamethasone, melatonin, avvastatin, and silymarin—can be incorporated into conduits to support nerve regeneration (Yang et al., 2022; Bianchini et al., 2023). For instance, melatonin, an endogenous neurohormone secreted by the pineal gland, can activate the Wnt/β-catenin pathway, inhibit SC apoptosis, promote SC proliferation, migration, and myelination, and reduce collagen accumulation and glial scar formation at injury sites. It is considered a promising treatment for PNI. Some biomaterials also have favorable electrical properties, such as conductivity or piezoelectricity, which can promote nerve regeneration and functional recovery by mimicking the electrophysiological signals of nerve cells. For example, Wu et al. (2017) showed that a polyaniline conductive hydrogel combined with nerve conduits and bioelectrical stimulation effectively promoted sciatic nerve regeneration in rats (**Figure 1**).

AI can enhance biomaterial design by predicting optimal properties such as porosity and degradation rate and by modeling the release of therapeutic agents. This integration supports the development of effective drug delivery systems and addresses the complex requirements of nerve repair.

## Application of Artificial Intelligence in Biomaterial Development

### Material design and optimization

Traditional biomaterial design methods are limited, with synthesis being difficult and time-consuming. The core advantage of AI lies in its superior data processing and analysis capabilities, which greatly improve research efficiency and accuracy. Using advanced algorithms, AI can rapidly extract valuable information from large datasets. ML models can analyze relationships among factors such as patient-specific nerve gaps, material degradation rates, nerve regeneration and maturation times, and material diameter and thickness. Through high-throughput computation, ML can predict material performance and guide the design of new materials (Stewart et al., 2020). For example, Chen et al. (2024c) used ML to study the correlation between structural information and gel properties of designed compounds, thereby predicting and guiding the design and synthesis of hydrogels. DL can be used for object detection, classification, and regression. Combined with DL models such as R-CNN and AlexNet, AI can rapidly analyze large volumes of materials data, identify the most promising candidates, and greatly shorten the discovery timeline for new materials (Evgeny et al., 2014). For example, Li et al. (2019) used DL to link the chemical and physical structure of gels with their self-assembly behavior, accelerating the discovery and design of novel hydrogel architectures.

Meanwhile, gene-editing technology—especially the CRISPR–Cas9 system—has become an important tool in biomedical research and therapy. In this approach, single-guide RNA (sgRNA) is designed to target specific sites in the gene of interest, directing the Cas9 protein to bind and cut DNA. In this process, sgRNA activity and off-target effects are key determinants of editing success. By analyzing large amounts of experimental data, ML algorithms can predict sgRNA activity and off-target effects, thereby optimizing sgRNA design and improving the accuracy and efficiency of gene editing and activation. For example, Listgarten et al. (2018) showed that using ML to predict sgRNA activity and off-target effects enables more efficient sgRNA design and improves editing accuracy. In addition, Kim et al. (2018) used DL to develop a fusion guide RNA (fgRNA) compatible with dead Cas9 (dCas9) and transcriptional activators, improving sgRNA activity prediction and enabling more precise gene targeting. Haeussler et al. (2016) evaluated multiple on-target and off-target scoring algorithms and integrated them into a CRISPR optimal rank prediction tool for sgRNA, using ML to optimize sgRNA design and improve the targeting efficiency and specificity of the CRISPRa system. As a derivative of CRISPR–Cas9, CRISPR activation (CRISPRa) has also been applied in nerve regeneration, where it can activate neurotrophic genes such as BDNF, GDNF, and NGF to create a more favorable microenvironment and promote neuronal survival, proliferation, and differentiation. Although CRISPRa, to some extent, avoids the off-target effects of gene editing, potential issues remain, such as nonspecific sgRNA binding that could aberrantly activate nontarget genes. Optimizing sgRNA design and targeting with ML can further reduce such risks. For example, a study used DL to optimize sgRNA design for two high-fidelity Cas9 variants, training models on large experimental datasets to predict sgRNA activity and off-target risk, thereby enabling more efficient sgRNAs and improving CRISPRa targeting accuracy (Wang et al., 2019). Thus, AI provides strong support for gene editing and activation technologies in nerve regeneration, helping researchers more precisely target specific genes and enhance the biological functions of nerve regeneration materials.

By analyzing large volumes of experimental and material-property data, AI can identify relationships between material properties and design parameters, thereby guiding optimization of mechanical properties, biocompatibility, and degradation rates. In 3D bioprinting, common challenges include low resolution, slow printing speed, and artifacts (Zhu et al., 2017). AI can optimize parameters such as printing speed and extrusion pressure through neural network algorithms, determine suitable material ratios and fabrication processes, and enable the creation of complex biomaterials with improved implantation accuracy and tissue-regeneration efficiency. For example, Huang et al. (2021) used 3D printing combined with ML to precisely adjust manufacturing parameters and achieve customized designs for nerve repair materials.

### Performance prediction and evaluation

AI, leveraging techniques such as artificial neural networks (ANNs) and quantitative structure–activity relationship models, can predict and evaluate the performance of biomaterials in terms of biocompatibility, mechanical stability, degradation behavior, and effects on neural regeneration. When combined with high-throughput screening, AI can deliver rapid and accurate evaluations, helping researchers identify new biomaterials that meet stringent criteria. For example, in medical biomaterials, AI, through ML and large-scale data analysis, offers powerful tools for predicting and evaluating material properties. Integrated with quantitative structure–activity relationship models, AI can rapidly predict the protein adsorption capacity and biocompatibility of biodegradable polymers (Niazi and Mariam, 2023), improving prediction efficiency and significantly reducing testing costs. By emulating the structure and function of biological neural networks, ANNs can model material properties and microstructure. For example, Gao et al. (2024) combined a trained ANN with a genetic algorithm to optimize the acoustic performance of resonant sound-absorbing metamaterials; the results showed that the ANN accurately predicted material properties and significantly improved optimization efficiency (Keshtegar et al., 2022).

With advances in computing hardware, convolutional neural networks (CNNs) have become a core DL architecture. CNNs can automatically extract multilevel features from images through multilayer convolution and pooling operations (Liu et al., 2025). These features include microstructural characteristics such as phase composition and pore distribution, enabling effective prediction of macroscopic material properties (de Mille et al., 2024). For instance, CNNs can analyze micrographs of rare-earth magnesium alloys to predict mechanical properties, including ultimate tensile strength, yield strength, and elongation (Cheng, 2024). In nerve regeneration materials, the NeuriteNet model developed by Vecchi et al. (2023) can accurately distinguish neurite growth under different substrate conditions via image analysis and correlates with quantitative metrics such as the neurite alignment index. Furthermore, research has shown that ML techniques can optimize surface topography features—such as groove height and spacing—to enhance nerve synapse growth (Payne et al., 2016). Together, these findings suggest that CNNs have significant potential in the design of nerve regeneration materials.

### Virtual experiments and applications

Animal studies have long been essential to the design and optimization of new materials. AI-driven virtual experiments provide a powerful alternative to traditional animal and *in vitro* tests, enabling virtual prototyping and iterative design (Dai et al., 2024). AI virtual cells combine AI with multimodal data to build precise, scalable virtual cell models capable of high-throughput simulation. Compared with traditional virtual cell models, AI virtual cells can more comprehensively capture cellular functions and, in some cases, replace laboratory experiments. This improves predictions of how materials interact with biological systems at the cellular level, reducing the need for extensive physical testing and streamlining development. AI can also enhance finite element simulations to model *in vivo* biocompatibility and mechanical behavior, predict treatment outcomes, lower costs, shorten R&D timelines, and reduce reliance on animal studies (Qian et al., 2025). For example, the elastic properties of hydroxyapatite/polymer composites can be evaluated using finite element methods, and running multiple simulations can optimize their mechanical performance (Shokrollahi et al., 2022). In parallel, ML can predict cell phenotypes and gene regulatory networks, further informing material selection (Okawa et al., 2016).

**Figure 2:**
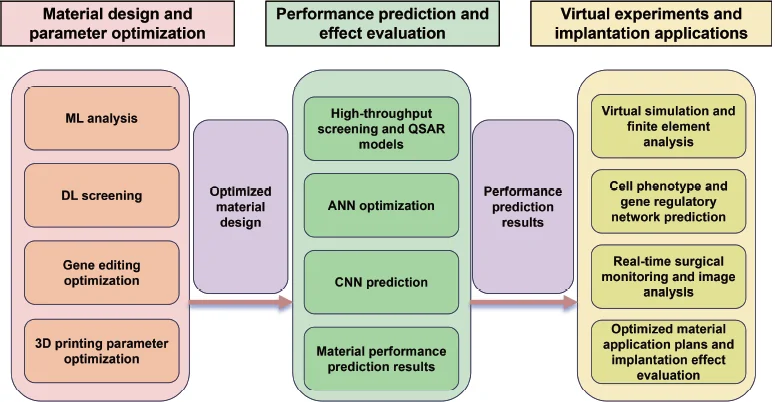
Application methods of artificial intelligence in the development of biological materials. The design phase includes machine learning analysis, deep learning screening, gene editing, and optimization of 3D printing parameters. These steps produce optimized material designs, whose performance is then predicted using high-throughput screening, QSAR models, ANN, and CNN. Finally, these predictions guide virtual experiments and applications, including simulation, cell phenotype prediction, real-time monitoring, and material application optimization. ANN: Artificial neural networks; CNN: convolutional neural network; DL: deep learning; ML: machine learning; QSAR: quantitative structure-activity relationship.

In addition, AI-driven image recognition has the potential to transform surgery by providing real-time monitoring and feedback during procedures (Sone et al., 2023). AI algorithms can track and analyze neural structures, dynamics, plasticity, and memory, and detect early signs of tissue damage or inflammation—valuable inputs for assessing material structure and performance (Ayuso et al., 2023). Advanced imaging and cell image analysis can quantify neuronal and synaptic growth. For instance, at nerve junctions, AI can perform ultrasonic detection, classification, and visualization, helping researchers better evaluate implant outcomes (Nozawa et al., 2023). AI can also monitor the long-term performance of implanted materials by analyzing follow-up imaging studies to ensure continued biocompatibility and function (Bonde et al., 2021). Additionally, AI enables high-precision detection and quantification of histological slide images. For example, Yang et al. (2019) used DL to analyze steatotic liver slides with high accuracy in disease identification, classification, and severity assessment. Beyond histology, AI can be applied to MRI, CT, and PET to provide a comprehensive view of a patient’s condition. By integrating multiple imaging modalities, AI can generate detailed 3D models of tissues and organs for surgical planning and training, improving diagnostic accuracy and enabling personalized treatment plans (Ahmed et al., 2024; **Figure 2**).

## Applications of Biomaterials in Peripheral Nerve Repair and Regenerative Medicine

### Development history of peripheral nerve repair materials

Over the past 30 years, the field of neural repair has advanced significantly, moving from a traditional reliance on autologous nerve transplantation to comprehensive treatment strategies that incorporate novel biomaterials and multiple advanced technologies. **Box 1** presents a timeline of these developments, highlighting key milestones and contributions that have shaped the field.

**Box 1:** Timeline and major events in the development of biomaterials for peripheral nerve repair

**Early stage (late 20th century to early 2000s)**
Late 1990s: Natural biomaterials (such as collagen, chitosan, and alginate) began to be explored for nerve repair (Jiang et al., 2022). 2001: Integra Lifescience Corporation’s Neurogen Nerve Guide received Food and Drug Administration (FDA) approval for the repair of peripheral nerve injuries. 2001: Collagen Matrix, Inc. received FDA approval for its collagen nerve cuff to repair peripheral nerve injuries. 2003: Polyganics BV received FDA approval for its neurolac nerve guide to repair peripheral nerve injuries.
**Material and technology integration stage (mid-2000s to early 2010s)**
2003: Polyganics BV's neurolac nerve guide has received FDA approval for the repair of peripheral nerve injuries. 2004: Lundborg et al. (2004) reported comparable outcomes between silicone tube bridging and traditional methods for repairing median and ulnar nerve defects in the forearm. 2007: Inada et al. (2007) successfully repaired digital nerve, superficial peroneal nerve, and facial nerve branch defects using polyglycolic acid (PGA)/collagen nerve conduits. 2010: Deumens et al. (2010) reviewed experimental strategies and future perspectives for peripheral nerve repair, emphasizing the importance of multifunctional materials.
**Composite and multifunctional materials development stage (2010s to early 2020s)**
2010: Deumens et al. (2010) reviewed experimental strategies and future perspectives for peripheral nerve repair, emphasizing the importance of multifunctional materials. 2012: Gu et al. (2012) used chitosan/PGA nerve conduits to repair a 30 mm median nerve defect in humans. 2018: Cacialli et al. (2018) studied the expression of brain-derived neurotrophic factor in the injured telencephalon of adult zebrafish. 2018: Chang et al. (2018) developed a spiral tissue-engineered nerve conduit for peripheral nerve regeneration.
**Artificial intelligence and biomaterials integration stage (2020s to present)**
2020: Chen et al. (2020) accelerated the discovery of all-natural plastic substitutes using machine learning. 2020: Stewart et al. (2020) studied the application of machine intelligence in nerve conduit design and production. 2021: Huang et al. (2021) investigated 3D-printed functional nerve conduits. 2023: Zhang et al. (2023) explored the use of deep learning to predict the mechanical properties of 316L stainless steel. 2024: Wang et al. (2024) developed a fully biodegradable and self-electrified device for neuroregenerative medicine. 2024: Vecchi et al. (2023) demonstrated the sensitivity of convolutional neural network image analysis to multifaceted measurements of neurite growth. 2025: Wang et al. (2025a) developed a biodegradable and restorative peripheral neural interface for the interrogation of neuropathic injuries.
Box 1 provides a comprehensive overview of the key milestones in the development of biomaterials for peripheral nerve regeneration, highlighting the continuous progress and innovation in the field.

**Figure 3:**
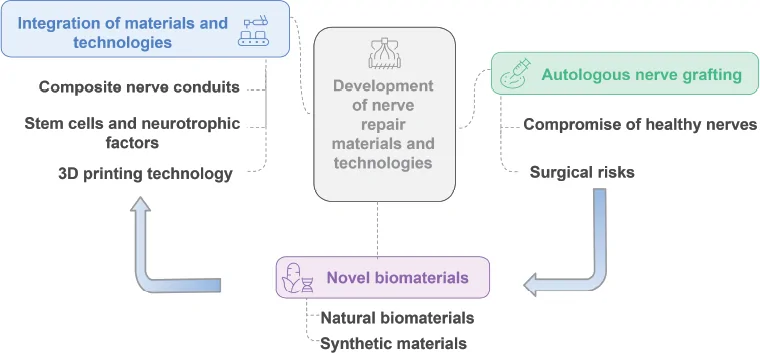
Development history of materials for peripheral nerve repair. The first stage is autologous nerve grafting, which faces challenges such as the compromise of healthy nerves and surgical risks. The second stage involves the development of novel biomaterials, including both natural and synthetic materials. The third stage is the integration of materials and technologies, which encompasses composite nerve conduits, stem cells, neurotrophic factors, and 3D printing technology. The arrows indicate the chronological order, starting with the limitations of autologous nerve grafting, moving through the development of novel biomaterials, and culminating in the integration of materials and technologies to advance the field of nerve repair.

These advancements listed in **Box 1** not only enhance the effectiveness of neural repair but also offer more possibilities for the treatment of nerve injuries in the future (**Figure 3**).

In the early days, autologous nerve transplantation, as the main method of nerve repair, required the transplantation of the patient’s own healthy nerves to the damaged area to promote nerve regeneration (Zhang et al., 2023). However, this method has many limitations. For instance, it necessitates sacrificing another healthy nerve, which may lead to structural damage and functional loss at the donor site. Additionally, the surgical process is complex and carries risks such as local infection and nerve mismatch (Zeng et al., 2022).

To overcome these limitations, researchers began to explore the application of new biomaterials. From the end of the 20^th^ century to the beginning of the 21^st^ century, natural biomaterials such as collagen, chitosan, chitin, alginate, agarose, and hyaluronic acid were gradually introduced into the field of neural repair (Jiang et al., 2022). Collagen is a gel-like substance extracted from animal skins that can promote wound healing. Due to its excellent biocompatibility, biological activity, and high expression in the ECM, collagen provides a supportive microenvironment for nerve regeneration after injury and is widely used in the preparation of nerve conduits (Zhang et al., 2024a, b). Chitosan and chitin are polysaccharides extracted from the shells of marine organisms, known for their good antibacterial properties, degradability, and strong cell adhesion characteristics (Zhao et al., 2017; Zhang et al., 2023). These properties enable them to better support cell growth and differentiation, making them ideal neural repair materials that have been widely studied and applied in tissue engineering (Yun et al., 2024). Meanwhile, alginates, agarose, and hyaluronic acid have been developed into hydrogels or scaffolds due to their excellent gel-forming and moisturizing properties, which support the growth and differentiation of nerve cells (Zhang et al., 2024a, b). The introduction of these materials has significantly improved the effectiveness of neural repair and reduced immune responses and biocompatibility issues. Additionally, synthetic materials such as polyglycolic acid (PGA), polylactic acid (PLA), and their derivatives, as well as poly(lactic-co-glycolic acid), have gradually entered the research process for neural repair at this stage due to their excellent mechanical properties, biocompatibility, and ease of processing (Tao et al., 2019; Yao et al., 2023b).

Although synthetic polymers can control their physical and chemical properties, they lack the biological activity and biodegradability of natural polymers, making them less likely to trigger inflammatory responses in the host. Therefore, they are suitable scaffold materials for implantation in the body (Lu et al., 2021). If the engineering advantages of synthetic materials can be combined with the biological activities of natural or modified biomaterials, more effective scaffolds that promote nerve regeneration and treat PNIs can be produced. Entering the 21^st^ century, the field of neural repair has further developed, leading to the integration of materials with materials and materials with technologies (Zhu et al., 2024a). For example, a composite neural conduit made from PLA and chitosan combines the excellent mechanical properties of PLA with the biological activity of chitosan, enabling better guidance for neural repair (Yi et al., 2024). By combining chitosan with natural and synthetic materials, researchers can fully leverage their complementary advantages to achieve improved biomechanical properties, chemical stability, and biocompatibility. Adjusting the ratio of chitosan to collagen in the composite material can also optimize its degradation rate in the body, providing suitable conditions for nerve regeneration (Yun et al., 2024). During this stage, bioactive materials combining stem cells and NTFs have been developed to promote neural regeneration. For instance, ECM was added to chitosan scaffolds, and structural alterations were made to address issues such as the slow degradation rate of chitosan materials themselves (Qi, 2023). Meanwhile, the emergence of degradable neural interface technology has provided new possibilities for long-term monitoring and repair of neural functions (Wang et al., 2025a).

**Figure 4:**
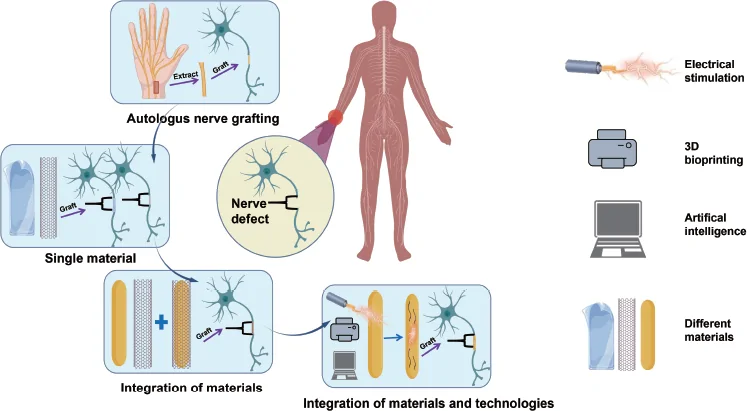
Mechanism of materials for peripheral nerve regeneration. The diagram illustrates four main stages in nerve repair: (1) autologous nerve grafting, where healthy nerve tissue is transplanted directly into the damaged area; (2) single material integration, using a biomaterial conduit to bridge the nerve defect; (3) integration of materials, combining various biomaterials to enhance repair outcomes; (4) integration of materials and technologies, utilizing advanced techniques such as 3D printing to create customized nerve conduits, and incorporating electrical stimulation to optimize nerve regeneration. This sequential development reflects the advancements and innovations in nerve repair technology, aiming to improve repair outcomes and patient recovery quality.

Additionally, the application of 3D printing technology enhances the complexity and functionality of neural repair materials. By adjusting printing parameters such as flow rate and pressure, the spatial structure—including porosity and surface roughness—can be precisely controlled. Additionally, biocompatible materials can be selected to rapidly manufacture complex peripheral nerve conduits (Tao et al., 2019). These conduits can be customized according to the specific needs of patients, thereby providing better support for the neural repair process (Zhang et al., 2022). Compared with autologous nerve grafts, 3D-printed conduits may promote axonal regeneration more effectively, reduce inflammatory responses, and accelerate the recovery of nerve function (Chen et al., 2020; Zhang et al., 2021a, b). The nanoparticle-enhanced nerve conduits prepared using 3D printing technology can promote nerve regeneration and functional recovery, with efficacy comparable to that of autologous transplantation (Tao et al., 2019; **Figure 4**).

With the emergence and application of advanced technologies such as AI, the structure and performance of PNR materials are continuously optimized, leading to ongoing improvements in the functionality of regenerated nerve tissues. As a result, artificial nerve grafts hold great promise for future applications (Inada et al., 2007).

### Current application in peripheral nerve diseases

#### Material loading substance

PNRMs provide the necessary support and signals for nerve regeneration by loading bioactive molecules and cells (Wang and Guo, 2015). These materials not only offer physical support for nerve regeneration but also create a favorable microenvironment for the survival and growth of nerve cells by releasing NTFs and other bioactive molecules. In the treatment of PNIs, artificial nerve grafts have gradually become an effective alternative to autologous nerve transplantation (Chang et al., 2025). Artificial nerve grafts offer several advantages, such as avoiding secondary damage, customizability, and precise regulation of bioactivity, which can better meet the therapeutic needs of patients. For instance, in the study by Wu et al. (2024), hydrogels loaded with NGF and BDNF were used. By controlling the release of these factors, a microenvironment conducive to nerve regeneration was created.

In addition, biomaterials loaded with SCs and neural stem cells can secrete NTFs and promote axonal growth. SCs play a key role in nerve regeneration; they can clear myelin debris and cell fragments at the injury site and secrete various neurotrophic factors, guiding the regeneration direction of nerve fibers to ensure accurate connections to target tissues. In polyneuropathy, especially diabetic neuropathy, researchers are exploring the use of hydrogels loaded with NTFs to improve the neural microenvironment and promote neural regeneration by controlling the release of these bioactive molecules (Yang et al., 2025). This strategy can provide continuous support for nerve regeneration, improve the nerve microenvironment, reduce inflammatory responses and tissue damage, thereby enhancing the effectiveness of nerve regeneration.

In autonomic neuropathy, the wearable device made of zwitterionic conductive hydrogel developed by Pan et al. (2025) has excellent conductivity and adhesion, allowing for reliable and comfortable assessment of the autonomic nervous system and real-time monitoring of heart rate variability parameters, providing support for the management of cardiac autonomic neuropathy in patients with diabetes (Pan et al., 2025). The emergence of this wearable device offers a new approach to monitoring and managing autonomic neuropathy. It can not only monitor relevant parameters in real time but also provide a long-term and comfortable monitoring experience for patients due to its conductivity and adhesion.

In the treatment of hereditary peripheral neuropathy, SCs are crucial for removing degenerated tissues and regenerating myelin for newly formed axons. Therefore, SCs from healthy nerves or different tissue sources have been transplanted into PNRMs to support myelin regeneration, representing a potential therapeutic approach (Yang et al., 2022). Meanwhile, gene therapy combined with biomaterials also shows promise. For example, using adeno-associated viral vectors to deliver NTFs, such as NT-3, can improve the neurotrophic environment and promote nerve regeneration. Furthermore, correcting gene mutations that cause diseases through gene editing technology, combined with providing physical support using biomaterials, can effectively promote nerve regeneration (Stavrou et al., 2021). Gene therapy, by introducing exogenous genes into nerve cells or supporting cells, can stably express NTFs or other beneficial proteins over the long term, thereby providing continuous support for nerve regeneration (Sulak et al., 2024). In addition, the application of gene editing technology has brought new hope for nerve regeneration. By correcting gene mutations that cause diseases and combining these approaches with biomaterials to provide physical support, nerve regeneration can be effectively promoted.

In the treatment of acquired peripheral neuropathy, researchers are exploring the use of hydrogels loaded with anti-inflammatory drugs to improve the neural microenvironment by inhibiting inflammatory responses and reducing tissue damage (Sanchez Rezza et al., 2022). The application of anti-inflammatory drugs can not only reduce inflammatory responses but also work synergistically with NTFs to further promote the survival of nerve cells and the growth of axons.

#### Guidance of nerve regeneration

PNRMs guide neural regeneration through their structure and physical properties, providing directionality and support for neural regeneration. For example, a porous structure is a key three-dimensional morphological feature of nerve guidance conduits (NGCs), which offers necessary pathways for the exchange and infiltration of cells, nutrients, signaling molecules, and the discharge of metabolic waste. By precisely tailoring pore size, porosity, and connectivity, fully permeable, semipermeable, and asymmetric NGCs can be constructed, providing new options for the treatment of long-segment peripheral nerve injuries (Wan et al., 2023). In cases of autonomic neuropathy, the amphoteric PNRMs developed by Pan et al. (2025) guide nerve regeneration through their structural and physical properties, providing directionality and support for nerve regeneration.

In the treatment of PNIs, silk protein nerve repair materials have been widely studied for PNR due to their excellent biocompatibility and designability (Mankavi et al., 2023). Silk protein has a porous structure, and its porosity and pore size can be adjusted, providing an ideal microenvironment for the attachment and growth of nerve cells. Through the bionic design of the ECM, its biocompatibility is further improved to promote the migration and differentiation of nerve cells (Lu et al., 2025). For example, the hydrogel form of polyvinyl alcohol simulates the endogenous ECM, providing an ideal microenvironment for nerve regeneration. Furthermore, some new types of neural catheter materials, such as polypyrrole, not only provide physical support but also offer electrical stimulation through loaded electrodes. This electrical stimulation can simulate neural electrical activities under physiological conditions, guiding the growth direction of nerve cells and promoting neural regeneration (Wang et al., 2022). The electrical conductivity of polypyrrole enables it to effectively transmit electrical stimulation. Meanwhile, its porous structure also facilitates the exchange of nutrients and the excretion of metabolic waste (Vijayavenkataraman et al., 2019). For sensory neuropathy, hydrogels loaded with BDNF promote the regeneration of sensory nerves by controlling the release of BDNF (Yu et al., 2024a, b). The softness and plasticity of hydrogels enable them to closely adhere to nerve tissues, providing a stable environment for the growth of nerve cells. Furthermore, the porous structure of the hydrogel facilitates the diffusion of nutrients and further supports nerve regeneration (Taisescu et al., 2025).

In addition to designing porous structures, constructing directional structures, such as microgrooves, is also an effective strategy. This design can guide nerve fibers to grow in a predetermined direction, remarkably improving the order and efficiency of nerve regeneration. It can also effectively reduce the dislocation of nerve fibers and false regeneration, further optimizing the effect of nerve repair. For example, Zhan et al. (2025) cleverly designed a multifunctional neural conduit by simultaneously embedding monolayer graphene and nanodiamond particles in polycaprolactone fibers, which can accurately guide the directional growth of nerve cells using its unique groove structure. At the same time, with the mechanical enhancement properties of nanodiamond and the high conductivity of single-layer graphene, the catheter can synergistically optimize the microenvironment of nerve regeneration, thus effectively promoting nerve regeneration.

#### Synergistic effects with other therapies

PNRMs can enhance the effect of nerve regeneration by combining with other treatment methods (Zhang et al., 2018; Song et al., 2025). For instance, photobiomodulation therapy activates photosensitive substances within cells through low-level laser treatment, promoting cell metabolism and energy production, and optimizing the microenvironment for nerve regeneration. Neural regeneration materials provide physical support for the attachment and growth of nerve cells through their porous structure and biocompatibility. The combined use of the two can not only enhance the biological activity of neural regeneration materials but also further promote the growth and functional recovery of nerve cells (Farazi et al., 2024; Zhang et al., 2024a, b).

In the treatment of motor neuropathy, the combined use of low-frequency electrical stimulation and biomaterials has been proven to significantly accelerate nerve repair. Jiao et al. (2021) reported that electrical stimulation can activate intracellular signaling pathways, promote axonal regeneration, and simultaneously optimize the microenvironment for nerve regeneration, reducing inflammatory responses. Biomaterials provide physical support for nerve cells through their porous structure and biocompatibility, while electrical stimulation further enhances the biological activity of these materials, promoting the growth and functional recovery of nerve cells (Lu et al., 2020; Chen et al., 2024a, b, d). Through electrical stimulation, the metabolic activity of nerve cells is enhanced, providing more adequate energy support for nerve regeneration. At the same time, electrical stimulation can also regulate the composition and structure of the extracellular matrix, creating more favorable conditions for the growth of nerve fibers. This multidimensional synergistic effect has remarkably improved the outcome of nerve regeneration.

Due to the limitations of cell metabolism, it is difficult to completely restore the structure and function of the nerve solely through the expansion and growth of new lateral buds in the vessel. The metabolic activity of nerve cells is relatively slow, especially during the repair process after injury, where the energy supply of the cells and the ability to clear metabolic by-products are both somewhat limited. This remarkably affects the speed and quality of nerve regeneration. Therefore, in recent years, research has begun to explore traditional Chinese medicine as an auxiliary means to enhance the repair effect of nerve conduits. Traditional Chinese medicine holds unique advantages in the field of nerve regeneration, as its various active components can promote nerve regeneration through different mechanisms. Lumbricus extract, for example, has been shown to greatly promote PNR as a traditional Chinese medicine preparation (Zhang et al., 2014a, b). Earthworm extract contains a variety of bioactive substances, such as lumbricin and lumbrokinase, which have anti-inflammatory, antioxidant, and cell proliferation-promoting effects. They can enhance the survival and regeneration capacity of nerve cells by regulating the metabolic activities of nerve cells (Zhu et al., 2024b).

In addition, extracts of Astragalus membranaceus, Angelica sinensis, and Genipin are also used to improve the nerve microenvironment due to their effects on blood circulation and inflammation, thus promoting nerve regeneration (Saito-Diaz et al., 2024). These traditional Chinese medicine extracts can improve the neural microenvironment through various pathways. For example, Astragalus membranaceus extract can enhance the immune function of the body and promote the repair of damaged nerves (Zhang et al., 2014a, b). Angelica sinensis extract has good anti-inflammatory and antioxidant effects, which can reduce the inflammatory response after nerve injury (Zhu et al., 2017). Genipin can promote the growth and differentiation of nerve cells by regulating intracellular signaling pathways (Wang and Wei, 2024). These studies demonstrate that integrating traditional Chinese medicine components with artificial nerve conduits can effectively compensate for the limitations of relying solely on conduits and further promote nerve regeneration and functional recovery. Traditional Chinese medicine components have the capacity to not only optimize the neural microenvironment but also bolster the regenerative potential of nerve cells through a variety of mechanisms. This multifaceted, combined therapeutic approach offers innovative concepts and practical methods for treating peripheral neuropathies.

To provide a comprehensive overview of the advancements in biomaterials for PNR, **Table 1** presents a comparative analysis of the new technologies and methods discussed in this section. This analysis includes descriptions, advantages, limitations, and specific application examples for each technology, highlighting their potential and challenges in the field of nerve regeneration.

**Table 1:** Comparative analysis of new technologies and methods for biomaterials in peripheral nerve repair

Technology/method	Description	Advantage	Limitation	Application example	Reference
Autologous nerve grafting	Transplanting healthy nerves to the patients to promote nerve regeneration.	Gold standard method	Sacrificing healthy nerves	Widely used in clinical applications	Chang et al., 2017
Biodegradable materials	Using biodegradable materials to create nerve conduits, reducing the need for secondary surgeries.	Avoid secondary surgeries	Difficult to precisely control degradation rate	PLA and PGA nerve conduits	Chang et al., 2018
Electrical stimulation	Using electrical currents to promote nerve regeneration and enhance axonal growth.	Promote axonal growth	Require external power sources	Conductive hydrogels	Wu et al., 2017; Park et al., 2020
PBM	Using low-level laser therapy to promote cell metabolism and nerve regeneration.	Non-invasive	Limited penetration depth	PBM combined with nerve conduits	Farazi et al., 2024
Multifunctional composite materials	Combining multiple biomaterials and functional components, such as neurotrophic factors and conductive materials, to enhance nerve regeneration.	Synergistic effects of multiple materials	Complex manufacturing processes	Chitosan/PGA nerve conduits	Gu et al., 2012
3D bioprinting	Using 3D printing technology to create customizable nerve conduits and scaffolds tailored to meet specific patient needs.	High-precision structural design	Require high technical expertise	3D-printed nerve conduits with vascular networks	Tao et al., 2019
AI-driven nerve regeneration technologies	AI at the core, optimizing and enhancing the performance of the above technologies, including material design, performance prediction, virtual experiments, and clinical applications.	Increase the efficiency and accuracy of material design	Require large amounts of high-quality data	AI-optimized 3D-printed nerve conduits	Wang et al., 2019; Chen et al., 2020; Huang et al., 2021

This comparative analysis offers a comprehensive overview of the key milestones in the development of biomaterials for peripheral nerve regeneration, highlighting the ongoing progress and innovation in the field. AI: Arterial intelligence; PBM: photobiomodulation; PGA: poly (glycolic acid); PLA: poly (lactic acid).

## Challenges and Future Directions

### Technical challenges

AI faces several challenges in the development of biomaterials. First, obtaining and integrating experimental data is difficult due to low data accuracy, the lack of standardized platforms, and the scarcity of high-quality clinical data, which results in insufficient model generalization ability. For example, when designing neural repair materials, one must consider the optimal dose of loaded NTFs, NTF mixtures, biological gradients, and other characteristics. The lack of relevant experiments limits the broad design and application of such materials (Klimaschewski et al., 2013). Second, the complexity of algorithms is high, especially when dealing with multi-omics data; more efficient AI algorithms are needed to cope with the depth and complexity of this data. In biomaterials research, although the AI simulation experimental environment and the results obtained are significant, more in-depth verification is still necessary. For example, some biomaterials that have been shown to promote the regeneration of blood vessels and nerve tissue in *in vitro* studies (Meyer et al., 2016; Rafique et al., 2023) may unexpectedly negatively affect the regeneration process *in vivo*. This discrepancy not only increases the complexity of the transition from AI simulation experiments to animal models but also complicates the further selection of materials from animal models for human trials.

In the field of biomaterials, in addition to the lack of relevant experimental data and the differences between *in vitro* and *in vivo* environments, many technical problems remain to be solved. First, the biocompatibility of biomaterials and the immune responses they may trigger are key issues. Some materials can provoke immune and inflammatory reactions when applied *in vivo*, hindering tissue repair and regeneration (Cui et al., 2022). Therefore, developing materials with better biocompatibility while reducing immune responses is an important direction for current research (Liu et al., 2019a, b). Second, the degradation rate and stability of biomaterials are also major technical problems. The degradation rate of biodegradable materials needs to match the rate of tissue regeneration. If it degrades too quickly, the material does not provide enough space for nerve regeneration; if it degrades too slowly, it may cause chronic inflammation. For example, the degradation rate of materials such as PLA and poly(lactic-co-glycolic acid) *in vivo* is difficult to control precisely (Yan et al., 2023). In addition, the mechanical properties of the material also need to match the target tissue. For example, materials used for nerve repair need to have sufficient strength and good flexibility while providing support and adjusting the shape and position of the material according to the nerve damage. The surface characteristics of materials also have important effects on their biocompatibility and cell adhesion ability. Furthermore, the stability, processability, biological activity, toxicity, and safety of biomaterials pose additional technical challenges. For instance, some hydrogel materials may lose moisture over long periods, resulting in the degradation of their mechanical properties, while some high-performance biomaterials may be difficult to process into complex structures (Lei et al., 2025). Moreover, materials must have certain biological activities to promote tissue regeneration and repair, such as loading growth factors or drugs to enhance their biological functions. They must also be non-toxic and safe when applied *in vivo*, as certain nanomaterials may accumulate in the body and cause toxic reactions. Therefore, a comprehensive safety assessment of the material is required.

In addition, the lack of interdisciplinary cooperation limits the development of technology. The development of biomaterials involves many fields, such as tissue engineering, 3D printing, genomics, and electronic information. For example, in the 3D printing process, NGCs coated with metal electrodes composed of nanoparticles are used as peripheral nerve interface devices to enhance signal propagation and thereby promote nerve regeneration (Wu et al., 2015). At the same time, stem cell technology has begun to integrate big data in the form of genomics, transcriptomics, proteomics, and ML to safely produce cells that are most suitable for the target patient and the regenerative process of interest (del Sol et al., 2017). Therefore, there is an urgent need to strengthen the synergy between various disciplines.

### Ethics and regulatory considerations

At the same time, biomaterials can advance technology, and the demand for personalized medical care is increasing, contributing to their gradual rise in popularity. Matching a specific neural graft to a patient is an important function of ML. However, during the implementation process, patient privacy protection and data security have become core issues. Due to the complexity and “black box” nature of ML algorithms, insufficient transparency may lead to biases and unexplainable decisions, thereby affecting treatment outcomes and patient trust. Therefore, the collection and analysis of personal data in scientific research activities using AI must strictly comply with regulatory and ethical principles, including informed consent, data anonymization, and transparency. Additionally, the improper use of AI technologies can lead to various ethical issues, such as the development of biological weapons and improper monitoring. Hence, scientific research institutions and enterprises need to establish ethical review mechanisms to ensure that AI applications comply with ethical standards. Researchers should receive ethical training to raise awareness of potential risks and prevent unethical research practices.

As medical devices, the production and marketing of new biomaterials must undergo a strict approval process. Currently, China’s National Medical Products Administration has clear and stringent requirements for the access of medical devices and implements full-process supervision over the manufacturing of nerve regeneration materials. For example, some 3D printing materials, such as PLA, deteriorate gradually under exposure, affecting print performance and product quality (Peng et al., 2024). Therefore, the National Medical Products Administration places special emphasis on the shelf life of nerve regeneration material products. This requires manufacturing enterprises to rigorously control the quality of raw materials and the production environment to ensure the stability and safety of the products.

In addition, establishing quality control standards is also a crucial aspect of ensuring the reliable application of AI technologies. However, at present, China has not yet formed a completely unified standardized production guideline for the manufacturing of nerve regeneration materials. Despite rapid technological developments, the advancement of relevant standards and norms lags behind in some areas. In practice, these gaps can lead to uneven product quality and potential risks to patient information security. To address these challenges, it is essential to strengthen the development and implementation of standardized protocols. This includes not only the creation of detailed production guidelines but also the establishment of a robust regulatory framework to oversee the entire process. Through these measures, we can ensure that products used in neuroregenerative medicine meet high-quality standards and that patients’ personal information remains secure and confidential. Continued standard-setting and regulatory efforts are vital to drive sustainability in this field and ultimately improve patient outcomes.

### Future prospects and innovations

With the continuous advancement of science and technology, AI has emerged as a key force in promoting the development of scientific research. Looking ahead, the application scope of AI will become wider, and its reshaping effect on scientific research will be more significant. From the construction of a macro-level scientific research ecosystem to the management and security enhancement of micro-level scientific data, as well as the improvement of AI model interpretability, these areas will become crucial focal points for the deep integration of AI and scientific research in the future.

Despite notable advancements in related fields, numerous pressing issues remain to be addressed. For instance, there is currently no gold standard for decellularization methods, and the assessment of immunogenicity in decellularized ECM scaffolds lacks unified criteria. Moreover, the degradation rates, biocompatibility, structural design, and optimization of preparation methods for synthetic stents have yet to be standardized. The challenges of processing difficulties and poor degradability in conductive materials also remain unresolved. Using graphene stents as an example, they provide unique advantages due to their high conductivity; however, the potential damage they may cause to surrounding tissues remains unclear. Additionally, there is an absence of clear and uniform standards for the selection of growth factors. These issues necessitate further research for resolution.

In the future, AI is expected to play a greater role in the development of biomaterials. By developing smarter algorithms and optimizing material design systems, it is possible to reduce the amount of data required while remarkably enhancing design efficiency. At the same time, AI can mine key information from massive experimental data and apply it to genomic analysis of various diseases (Zhang et al., 2021a, b). For example, Zhang et al. (2023) found through whole-genome sequencing that about 20% of methylated DNA fragments change methylation levels after PNIs, and these changes are closely related to axonal regeneration ability. Therefore, in the future, basic mechanism research can be advanced by enriching ML algorithms and combining genome sequencing and other methods, thereby accelerating the development of neural regeneration and repair materials.

In the field of personalized therapy, establishing standardized data platforms has become a key step. These platforms are designed to collect a large amount of sample clinical data from different patient populations. By integrating such extensive information, physicians can develop highly accurate and tailored treatment plans for each patient to meet their specific medical needs. The data collected includes detailed patient histories, diagnoses, and treatment outcomes, all of which support a comprehensive understanding of an individual’s health status. Additionally, the deep convergence of AI and biomaterial development will revolutionize the field of neuroregenerative medicine. Advanced DL algorithms meticulously analyze medical images and biological data. These algorithms can detect subtle patterns and anomalies that may not be visible to the human eye, providing a more accurate basis for diagnosis and treatment. As a result, progress in neuroregenerative medicine has accelerated significantly, improving patient outcomes and quality of life. This integration not only optimizes the therapeutic process but also paves the way for the development of innovative biomaterials capable of supporting nerve regeneration and repair.

## Safety Considerations

### Adverse reactions and toxicity

In the treatment of peripheral nerve diseases, while the application of biomaterials has raised many hopes, it has also led to certain adverse and toxic reactions (Minev et al., 2015). First, as an alternative therapy to autologous nerve transplantation, the implantation of exogenous biomaterials into the human body may trigger an immune response, resulting in inflammatory or allergic reactions. Severe inflammatory responses can lead to the death of SCs at the injury site and the formation of scars, which complicates the process of nerve regeneration (Zheng et al., 2020). For instance, certain biomaterials may be recognized by the body as foreign substances, activating immune cells and releasing inflammatory factors, thereby causing local inflammatory responses such as redness, swelling, and pain (Kasravi et al., 2023).

Furthermore, some biomaterials may also trigger persistent and intense immunosuppressive phenomena. For example, components of biomaterials such as microparticles and nanoparticles can release potent anti-inflammatory cytokines, such as interleukin-10 and transforming growth factor-β, and recruit major regulatory immune cells, such as M2 macrophages. This can lead to immunosuppression and affect the normal immune function of the organism (Boraschi et al., 2017). Zhang et al. (2021a, b) have reported that the surface properties, chemical composition of materials, and the microenvironment of the implantation site all influence the occurrence and extent of inflammatory responses.

Additionally, regarding material safety, side effects may also arise during the degradation process after material implantation. The degradation products may cause toxic damage to surrounding tissues and negatively affect the microenvironment for nerve regeneration (Yi et al., 2019). Ideally, the degradation rate of the material should be matched with the rate of regeneration. If the degradation is too rapid, it cannot provide adequate support; if it is too slow, it may induce rejection reactions. For instance, PLA is a commonly used biodegradable material. Its degradation product, lactic acid, may lead to local acidosis and disrupt the microenvironment for nerve regeneration. This acidic environment not only inhibits the growth and differentiation of nerve cells and induces cell apoptosis but may also further trigger inflammatory responses and hinder nerve regeneration (Lu et al., 2021).

### Material evaluation and safety protocols

In the treatment process for peripheral nerve diseases, the safety of biomaterials is the primary consideration. Additionally, the effectiveness of the materials must also be taken into account during their application. The materials should possess sufficient mechanical support capacity and be compatible with surrounding tissues to maintain the openness of the nerve regeneration channels (Gu et al., 2011; Jiang et al., 2021). Furthermore, the materials should have specific structural characteristics, such as designed porosity and catheter pore size, to facilitate the attachment and migration of regenerated nerve cells, provide guidance for new nerves, and create a suitable regeneration microenvironment for nerve regeneration (Kong et al., 2022).

Although the use of AI technologies to assist in the treatment of peripheral nerve diseases holds great potential, it also faces challenges such as technical reliability and privacy security. For prediction models based on AI, the singularity of the evaluation index may result in varying material simulation effects (Kerner et al., 2021). Moreover, the resolution and contrast of image acquisition by AI, as well as the accuracy of algorithm analysis, will all affect the reliability of disease diagnosis and treatment (Litjens et al., 2017). Privacy and security are crucial for safeguarding patients’ rights and interests. Therefore, when constructing relevant clinical databases, it is essential to strictly adhere to applicable laws and regulations to ensure the privacy rights of patients’ information and prevent data leakage and abuse.

## Clinical Transformation and Regulatory Landscape

### Clinical trial protocols registered with ClinicalTrials.gov database

The ClinicalTrials.gov database is an authoritative platform for clinical research information, containing numerous ongoing or completed clinical trials. It provides transparent and comprehensive study details for researchers, clinicians, and patients, promoting the standardization and transparency of medical research while offering patients access to the latest treatments. Within ClinicalTrials.gov database, we searched using “Peripheral Nerve Injuries” as the “Condition/Disease” keyword and “conduit” as the “Intervention/Treatment” keyword, retrieving seven related clinical trials, as shown in **Box 2**.

**Box 2:** Relevant clinical trial protocols registered with ClinicalTrials.gov database

**Observational studies**
(1) NCT03359330 (January, 2018–December, 2021, unknown status, all others [individuals, universities, organizations]). Mid-term effect observation of biodegradable conduit small gap tublization repairing peripheral nerve injury. (2) NCT01526681 (November, 2008–December, 2025, Recruiting, Industry). RANGER® Registry of Avance® Nerve graft’s utilization and recovery outcomes post peripheral nerve reconstruction. (3) NCT04788030 (January, 2008–January, 2021, completed, all others [individuals, universities, organizations]). Reconstruction of digital nerve lesions with muscle-in-vein conduits (4) NCT01573650 (March, 2015–July, 2021, completed, all others [individuals, universities, organizations]). Optimization of peripheral nerve reconstruction: a non-inferiority trial.
**Interventional studies**
(1) NCT06529835 (September, 2024–August, 2028, not yet recruiting, Industry). NeuroSpan-1: A comparison of neurospan bridge, neuragen nerve guide, and nerve autograft for peripheral nerve repair. (2) NCT00948025 (June, 2009–December, 2014, Terminated, Industry). CHANGE: A comparative post-marketing study of commercially available peripheral nerve gap repair options. (3) NCT02970864 (August, 2017–May, 2021, completed, all others [individuals, universities, organizations]). UMANC: A phase I trial of a novel synthetic polymer nerve conduit ‘polynerve’ in participants with sensory digital nerve injury.

These experiments cover a variety of research types and designs, including observational and interventional studies, reflecting the diversity and innovation of current PNR. Among them, NCT03359330 and NCT04788030 belong to observational studies, primarily providing preliminary references and guidance for clinical practice by observing the effects of specific repair methods or materials in practical applications. In contrast, NCT06529835 and NCT00948025 are interventional studies that compare the effects of different repair methods or materials, verifying their effectiveness and safety. These trials also adopted a comparative study design; for example, NCT06529835 compared the effects of three PNR methods: NeuroSpan Bridge, NeuraGen Nerve Guide, and nerve autologous transplantation. This design intuitively evaluates the advantages and disadvantages of various methods or materials, providing a stronger basis for clinical practice.

Additionally, the funding sources for these experiments include both the industrial and non-industrial sectors. A comparison reveals that research funded by the industry often focuses on the development and application of neural repair products, yielding results that remarkably promote related products. Conversely, research funded by non-industry sectors tends to focus more on academic inquiries, offering broader references for future clinical practice.

Moreover, the time span of the trials varies from several years to over a decade. Long-term research design helps to comprehensively assess the long-term effects and potential problems of the repair methods; however, it may also lead to challenges such as patient loss to follow-up and technological updates. Furthermore, these experiments involve various PNR materials and techniques, such as biodegradable conduits, nerve transplantation, synthetic polymer nerve conduits, and muscle-venous conduits. This indicates that the field of PNI repair is continuously exploring and applying new materials and techniques to enhance the effectiveness and safety of PNR.

The clinical trials registered with ClinicalTrials.gov database that were included in this review are listed in **Box 2** collectively aim to explore and optimize various methods and materials for PNR. These studies cover the assessment of new nerve repair materials (e.g., biodegradable conduits and synthetic polymer nerve conduits such as “Polynerve”), long-term tracking of commercial nerve grafts (e.g., Avance®), and direct comparisons between different repair techniques (e.g., NeuroSpan Bridge, NeuraGen Nerve Guide, and nerve autografts). Additionally, they include observational studies on specific techniques (e.g., muscle-in-vein conduits for digital nerve repair) and post-marketing comparisons of available nerve repair options. By combining observational and interventional approaches, these trials evaluate the safety and efficacy of novel materials and techniques, while also providing comprehensive information on existing repair solutions. This integrated research effort is crucial for enhancing clinical practice, driving innovation in nerve repair technologies, and ultimately improving patient outcomes and quality of life.

### U.S. Food and Drug Administration–approved peripheral nerve repair materials

The U.S. Food and Drug Administration (FDA) plays a key role in the approval and regulation of medical devices in the United States. Its rigorous approval process ensures the safety and effectiveness of medical devices, providing essential protection for patients. The FDA’s comprehensive evaluation framework includes extensive preclinical testing, clinical trials, and post-market surveillance designed to maintain high-quality and safety standards for medical products. In recent years, the FDA has approved a range of PNR materials, reflecting continued progress in the field. These approvals are based on substantial evidence that these materials promote neural regeneration and functional recovery. Approved materials include innovative stents, catheters, and bioactive compounds that have shown potential in both laboratory settings and clinical applications. These advances expand treatment options for patients with peripheral nerve injuries and offer new hope for improved outcomes. Details of some of the approved devices are summarized in **Box 3**, which provides their names, manufacturing companies, intended uses, and the biological materials associated with them.

**Box 3:** FDA-approved peripheral nerve repair materials

**Poly(DL-lactide-ε-caprolactone)**
(1) Neurotube (Neuroregen LCC, March 22, 1999, K983007). Single use in patients with a peripheral nerve injury where the nerve gap is more than or equal to 8 mm but less than or equal to 30 mm. (2) Neurolac nerve guide (Polyganics BV, October 10, 2003, K032115; May 04, 2005, K050573; October 20, 2011, K112267) Reconstruction of a peripheral nerve discontinuity up to 20 mm.
**Collagen**
(1) Neurogen nerve guide (Integra Lifescience Corporation, June 22, 2001, K011168). Repair of peripheral nerve discontinuities. (2) Collagen nerve cuff (Collagen Matrix, Inc., September 21, 2001, K012814). Repair of peripheral nerve discontinuities. (3) Flexible collagen nerve cuff (Collagen Matrix, Inc., April 3, 2014, K131541). Management of peripheral nerve injuries in discontinuities. (4) Neurogen 3D (Integra Lifescience Corporation, April 24, 2014, K130557). Repair of peripheral nerve discontinuities.
**Polyvinyl alcohol**
(1) SaluMedica nerve cuff (SaluMedica, LLC, November 24, 2000, K002098). Repair of peripheral nerve discontinuities.
**Pure collagen membrane:**
CoVa ORTHO-NERVE (Biom'Up S.A., March 2, 2012, K103081). Repair of peripheral nerve injuries in which there is no gap or where gap closure can be achieved by flexion of the extremity.
**Chitosan:**
(1) Reaxon Plus (Medovent GmbH, December 2, 2015, K143711). Repair of peripheral nerve discontinuities up to 10 mm. (2) NeuroShieldTM (Monarch Bioimplants GmbH, May 31, 2019, K190246). Repair of peripheral nerve injuries in which there is no gap or where gap closure can be achieved by flexion of the extremity.
**Polyglycolic acid and collagen (porcine skin):**
Nerbridge (Toyobo Co., Ltd., May 20, 2016, K152967). Repair of peripheral nerve injuries where there is no gap or where gap closure can be achieved by flexion of the extremity.
**Calcium alginate and hyaluronic acid:**
VersaWrap nerve protector (Alafair Biosciences, Inc., September 14, 2020, K201631). Management of peripheral nerve injuries in which there has been no substantial loss of nerve tissue.
**Bioabsorbable extracellular collagen matrix derived from small intestinal submucosa with nickel-titanium alloy:**
Nerve tape (BioCircuit Technologies, Inc., July 15, 2022, K210665). Repair of peripheral nerve discontinuities where gap closure can be achieved by flexion of the extremity.
**Poly(lactide-co-caprolactone):**
Rebuilder nerve guidance conduit (CelestRay Biotech Company, LLC, January 29, 2024, K230794). Reconstruction of a peripheral nerve discontinuity up to 20 mm.
**Polyglycolic acid:**
Mochida nerve cuff (Mochida Pharmaceutical Co., Ltd., June 21, 2024, K233322). Repair of peripheral nerve discontinuities where gap closure can be achieved by flexion of the extremity/management of peripheral nerve injuries.

These materials have made significant progress in design, material selection, and clinical application. In terms of material types, the development of new polymer materials has become a hot topic in recent years. Traditional biological repair materials, such as the collagen used in the Neurogen Nerve Guide and collagen nerve cuff, often struggle to provide continuous mechanical support and are mostly used for nerve injuries without substantial defects. In recent years, a variety of new biomaterials have emerged. For instance, polyvinyl alcohol used in the SaluMedica Nerve Cuff and poly(DL-lactic acid-ε-caprolactone) used in the Neurotube are both high-molecular-weight polymer materials that meet biocompatibility standards. They demonstrate good biocompatibility, low immunogenicity, and degradability in neural repair, while also providing stable mechanical support during the process of neural regeneration. Similarly, the Neurolac Nerve Guide also utilizes this material.

Additionally, some new materials not only serve the function of repairing nerves but also combine other properties. The VersaWrap Nerve Protector combines calcium alginate and hyaluronic acid, offering good biocompatibility and mechanical properties suitable for nerve protection. Nerve tape combines a bioabsorbable extracellular collagen matrix with nickel-titanium alloy, providing good flexibility and strength, making it suitable for nerve repair.

In the future, by integrating AI and ML technologies, the design and development of neural repair materials will become more intelligent. Through high-throughput screening and simulation experiments, the design and performance of materials can be rapidly optimized, leading to the development of materials more suitable for nerve regeneration. Meanwhile, as mentioned above, future materials will not only be limited to nerve repair but will also incorporate additional functions such as electrical stimulation, optogenetic stimulation, and drug release. Determining how to combine these methods to synergistically enhance nerve regeneration performance is a key research direction for future nerve repair materials.

**Box 3** lists FDA-approved materials for PNR, highlighting their names, manufacturers, approval dates, 510(k) numbers, indications for use, and composition materials. These devices are designed to address various peripheral nerve injuries, with specific indications ranging from repairing nerve discontinuities to reconstructing nerve gaps of certain lengths. The materials used include a variety of biocompatible substances such as poly(DL-lactide-ε-caprolactone), collagen, polyvinyl alcohol, chitosan, and others. These approvals reflect the regulatory endorsement of these materials for specific clinical applications in PNR, providing clinicians with a range of options to choose from based on the nature and severity of nerve injuries.

### Material evaluation and regulatory framework

The funding projects of the National Institutes of Health (NIH) in the field of biomaterials and peripheral nerve regeneration (PNR) have achieved remarkable results in recent years, promoting the development of basic research and providing new ideas and methods for clinical applications. These projects and related papers demonstrate the influence and future development directions of this field (**Box 4**).

**Box 4:** Projects funded by the National Institutes of Health

**Surgical sealants and adhesives**
(1) R01HL140618: Engineering highly elastic surgical sealants with hemostatic properties (NHLBI, 2018–2023, University of California, Los Angeles). Outputs: Photocrosslinkable gelatin/tropoelastin hydrogel adhesives for peripheral nerve repair. (2) R01EB023052: Engineering a naturally derived and highly adhesive surgical sealant (NIBIB, 2018–2024, University of California, Los Angeles). Outputs: Photocrosslinkable gelatin/tropoelastin hydrogel adhesives for peripheral nerve repair.
**Nerve guidance conduits**
R01NS094218: Focal sustained release chemotherapy-loaded biomaterials at tumor sites (NINDS, 2017–2021, Tufts University, Medford). Outputs: Nerve guidance conduits with hierarchical anisotropic architecture for peripheral nerve regeneration.
**Stem cell therapy and regeneration**
(1) F32HD098808: Enhanced stem cell therapy with rehabilitation strategies for peripheral nerve regeneration (NICHD, 2019–2021, Stanford University). Outputs: Elastin-like proteins to support peripheral nerve regeneration in guidance conduits. (2) T32GM119995: Graduate training in stem cell biology and regenerative medicine (NIGMS, 2017–2022, Stanford University). Outputs: Elastin-like proteins to support peripheral nerve regeneration in guidance conduits.
**Tissue mimics and injectable delivery**
F31HL150942: Creating hydrogel tissue mimics to better understand congenital heart disease (NHLBI, 2020–2021, University of Florida). Outputs: Decellularized peripheral nerve as an injectable delivery vehicle for neural applications.
Box 4 presents NIH-funded projects on PNR and regeneration. These projects encompass surgical sealants, adhesive sealants, chemotherapy biomaterials, stem cell therapies, and hydrogel tissue mimics. They are supported by the NHLBI, NIBIB, NINDS, NICHD, and NIGMS, producing outputs such as photocrosslinkable adhesives and nerve conduits. The projects are conducted at various renowned universities. These NIH-funded initiatives demonstrate a commitment to advancing biomedical research in nerve repair and regeneration, with potential applications in clinical settings.

For instance, the two projects, “Development of Highly Elastic and Hemostatic Surgical Sealants” (R01HL140618) and “Research and Development of High-Viscosity Surgical Sealants from Natural Sources” (R01EB023052), both focus on the development and application of photocross-linked gelatin/protoelastin hydrogel adhesives. Both projects not only proved the effectiveness of the adhesive in PNR but also demonstrated its broad application potential in various surgical operations. Meanwhile, the application of elastin-like proteins in the project “Biomaterials for Locally Sustainably Released Chemotherapy Drugs at Tumor Sites” (R01NS094218) and the project “Stem Cell Therapy Combined with Rehabilitation Strategies to Enhance Peripheral Nerve Regeneration” (F32HD098808) reflects the importance of comprehensive treatment methods in nerve regeneration (Lu et al., 2021). Furthermore, the project “Postgraduate Training in Stem Cell Biology and Regenerative Medicine” (T32GM119995) has promoted the application research of elastin-like proteins in neural conduits by cultivating a new generation of research talents. These projects not only provide significant support for basic research in the field of regenerative medicine but also offer a solid talent foundation for future innovations. Through interdisciplinary training, this project provides new methods and ideas for solving complex medical problems. Finally, the project “Creating Hydrogel Tissue Mimics to Better Understand Congenital Heart Disease” (F31HL150942) developed acellular peripheral nerves as an injection nerve repair carrier, demonstrating an innovative approach to developing biomaterials by leveraging the natural properties of nerve tissues.

To sum up, these projects funded by the NIH have made remarkable progress in the fields of biomaterials and PNR. They have achieved breakthroughs in basic research and demonstrated great potential in clinical applications. These research achievements not only bring new hope to the field of neural repair but also provide important references and lessons for research in related areas. In the future, with the further advancement of these projects and the launch of new ones, it is expected that more effective treatment options will be available for patients with nerve injuries, significantly improving their quality of life.

### Classic clinical trials and outcomes

Nerve repair stents can be divided into nonabsorbable and absorbable stents based on their material characteristics and intended use. Nonabsorbable stents are typically made of durable materials that can remain in the body for an extended period, providing long-lasting support and stability. Absorbable stents are designed to degrade and be absorbed by the body over time, thereby reducing the risk of long-term complications and the need for additional surgery. Testing of nonabsorbable stents focuses on long-term outcomes, such as the prevention of restenosis and the durability of stent materials. In contrast, absorbable stent testing emphasizes the biocompatibility and degradation characteristics of the materials used. We list five classic clinical trials based on this classification. These trials have significantly improved our understanding of the efficacy and safety of nerve repair stents. In the table, the research content of each trial, the application scope of the stent, and relevant experimental results are discussed (**Box 5**).

**Box 5:** Representative clinical trials

**Single-material scaffolds**
(1) Silicone tube bridging versus traditional method (Lundborg et al., 2004). Scaffold type: single material (silicone tube). Primary research focus: repair of ulnar/median nerve defects in the forearm. Trial design: randomized controlled clinical trial, 5-year follow-up. Main results: comparable repair outcomes; silicone tube group had milder cold intolerance symptoms. Conclusion: Silicone tubes are effective for nerve repair. (2) Expanded polytetrafluoroethylene (ePTFE) tube for ulnar nerve repair (Stanec et al., 1998). scaffold type: single material (ePTFE). Primary research focus: repair of a 29 mm ulnar nerve defect. Trial design: clinical case report. Main results: good recovery of sensory and motor functions. Conclusion: ePTFE tubes are effective for ulnar nerve repair. (3) Polyglycolic acid (PGA) nerve guide for digital nerve injury (Weber et al., 2000). Scaffold type: single material (PGA). Primary research focus: repair of digital nerve injuries. Trial design: randomized, controlled, multicenter clinical trial, 3–12 months follow-up. Main results: for nerve defects within 4 mm, PGA tube repair resulted in better sensory recovery than end-to-end neurorrhaphy. Conclusion: PGA tubes are effective for short-distance nerve defect repair.
**Composite material scaffolds**
(1) Chitosan/PGA composite for upper limb nerve defects (Fan et al., 2008). Scaffold type: composite material (chitosan and PGA). Primary research focus: repair of 30–35 mm upper limb nerve defects. Trial design: clinical application, 3-year follow-up. Main results: significant recovery of limb function, reaching good to excellent levels. Conclusion: Composite materials are effective for long-distance nerve defect repair. (2) PGA/collagen composite for various nerve defects (Inada et al., 2004, 2005, 2007). Scaffold type: composite material (PGA/collagen). Primary research focus: repair of digital nerve, superficial peroneal nerve, and facial nerve branch defects. Trial design: clinical application. Main results: successful repair of nerve defects, good functional recovery. Conclusion: PGA/Collagen composites are effective for various nerve repairs.
Box 5 outlines representative clinical trials on nerve repair, examining various scaffold materials and their effectiveness. The trials include comparisons of silicone tubes to traditional methods, ePTFE tubes for ulnar nerve repair, PGA tubes for digital nerve injuries, and composite materials (chitosan/PGA and PGA/collagen) for longer nerve defects. These studies demonstrate the efficacy of different materials in repairing nerve defects, with results indicating significant functional recovery and improved sensory and motor functions.

Among them, nonabsorbable stents are represented by silicone tube catheters. For instance, Lundborg et al. (1997, 2004) compared the repair effects of silicone tube bridging and traditional methods on forearm ulnar nerve/median nerve defects through a randomized controlled clinical trial. The results showed that the repair effects of the two were comparable. Additionally, Stanec and Stanec (1998) used ePTFE catheters to repair 29 mm ulnar nerve defects and achieved good recovery effects in sensory and motor functions. These studies indicate that nonabsorbable stents have certain advantages in neural repair, particularly in the repair of short-distance neural defects, as they provide stable physical support and effectively promote neural regeneration. Research on absorbable scaffolds is more extensive and includes both single materials (such as PGA, collagen, and chitosan) and composite materials (such as PGA/collagen and chitosan/PGA). The randomized, controlled, multicenter clinical trial by Weber et al. (2000) demonstrated that for finger nerve injuries with nerve defects within 4 mm, sensory recovery after PGA nerve catheter repair was superior to that of traditional end-to-end sutures. Meanwhile, the clinical application of chitosan/PGA composite materials, developed by Gu et al. (2012) for repairing 30–35 mm upper limb nerve defects, showed significant recovery of limb function in patients after a 3-year follow-up, reaching an excellent level. Japanese scholars Inada et al. (2004, 2005, 2007) successfully repaired defects in the common finger nerve, superficial peroneal nerve, and anterior branch of the facial nerve using PGA/collagen catheters with built-in collagen sponges in the lumen. They also achieved relative success in repairing nerve defects after neuroma resection. These studies not only demonstrate the potential of absorbable scaffolds in nerve repair but also highlight that composites have unique advantages in addressing long-distance nerve defects. Specifically, absorbable stents have shown promising results in reducing the risk of long-term complications associated with permanent implants, such as chronic inflammation and mechanical stress. Their gradual degradation facilitates natural tissue integration and minimizes the need for additional surgical intervention. Meanwhile, composites that combine different biocompatible components serve as an effective solution for bridging a wide range of neural gaps. These materials leverage multiple substances to promote nerve regeneration and functional recovery over longer distances. The synergistic effect of these combined materials enhances the overall repair process, providing better outcomes for patients with severe nerve damage. As research in this field progresses, the development of next-generation neural repair scaffolds is expected to further optimize these advantages and ultimately improve the quality of life for individuals affected by nerve damage.

## Limitations

Although this article provides a comprehensive discussion on the application, challenges, and prospects of AI in the development of materials for PNR, there are still some deficiencies. First, regarding literature retrieval, despite using a relatively extensive search strategy, we were constrained by time and resources, leading to the use of only one database, Web of Science. This may result in the omission of some important studies that could have been included in the analysis. Furthermore, considering the rapid development of the research field, some of the latest research achievements may not be included in a timely manner, which might affect the comprehensive understanding of the current research progress.

Second, while discussing the applications of AI technologies in this paper, although its potential in material design, performance prediction, and virtual experiments is emphasized, the exploration of specific technical details is not sufficiently in-depth. For example, this review paper does not provide a detailed analysis of the accuracy and reliability of AI technologies when handling complex biomedical data, nor does it offer solutions for further optimizing these algorithms to improve prediction accuracy. Additionally, this article fails to conduct a more detailed classification and discussion of the specific application differences of AI technologies in various types of peripheral nerve diseases, such as diabetic neuropathy and hereditary neuropathy.

Third, when addressing ethical and supervisory issues, although the importance of patient privacy protection and data security is mentioned, the article does not provide more operational suggestions on how to balance the relationship between technological innovation and ethics in practical applications. Moreover, the discussion of the regulatory and approval challenges that AI technologies may face during the clinical transformation process is relatively brief and does not deeply analyze how to promote the clinical application of related technologies within the regulatory frameworks of different countries and regions.

Finally, while looking forward to future development directions, this paper proposes the potential for deep integration of AI and biomaterials development but fails to provide more explicit suggestions on the specific mechanisms and steps for achieving interdisciplinary cooperation. Furthermore, regarding the application prospects of AI technologies in personalized medicine, although its significance is emphasized, this article does not offer a specific implementation path for achieving precise treatment in actual clinical scenarios. These limitations provide further avenues for exploration in future research in related fields, particularly in deepening technical details, improving the ethical framework, and promoting clinical transformation.

## Conclusions and Prospects

In conclusion, AI has demonstrated great potential in the development of PNR materials. By leveraging ML and DL capabilities, AI plays a significant role in material design and performance prediction. By analyzing and providing feedback on material performance during simulation experiments, AI can predict and optimize material outcomes. This not only promotes the regeneration and myelination of nerve fibers but also regulates cell signal transduction, thereby guiding the development of neural regeneration biomaterials more effectively. The focus on intelligence, personalization, and multi-functionality aims to achieve more efficient repair of PNI. These technologies can enhance the efficiency of material development and provide more precise and effective solutions for clinical treatment, significantly improving the prognosis and quality of life for patients with PNI.

Compared with previous studies, this review offers a more holistic view by integrating the latest advancements in AI with their clinical implications and ethical considerations. While prior reviews have primarily concentrated on the technical aspects of AI in material science, this review uniquely addresses the challenges and future directions in the context of neural regeneration. For example, it highlights how AI can optimize the design of NGCs by analyzing large datasets of material properties and predicting their performance in clinical settings. This approach provides a fresh perspective on how AI can bridge the gap between laboratory research and clinical practice, emphasizing the need for interdisciplinary collaboration and ethical oversight. The scientific value of this review lies in its detailed analysis of the current state of AI in PNR materials, identifying key limitations and proposing innovative solutions. The findings are universally applicable, addressing fundamental challenges in material design, data security, and clinical integration that are relevant across various fields of biomedical engineering. By emphasizing the importance of big data and interdisciplinary cooperation, this review provides a roadmap for researchers and clinicians to develop more effective and personalized treatments for PNI. Future research should focus on optimizing AI algorithms and material design to achieve more precise neural repair strategies, ensuring that these advancements translate into tangible benefits for patients.

However, despite the advancements AI has made in the development of neural regeneration materials, it still faces numerous challenges. Regarding security issues, although AI has been widely used in various fields, data and models remain exposed to security risks during the training and execution phases. The performance of ML systems relies heavily on training data, and adversarial samples and data poisoning attacks can lead to system misjudgment or reduced accuracy. Model attacks, including backdoor attacks, model theft, reverse attacks, and membership inference attacks, can reveal sensitive information or steal model parameters. Despite the existence of multiple defense technologies, new attack models are emerging, creating an urgent need to develop systems that remain robust against malicious attacks.

Furthermore, in the field of neural regeneration, the acquisition and integration of experimental data are challenging; there is a lack of standardized platforms, and high-quality clinical data is scarce, resulting in insufficient generalization ability of the models. Additionally, the algorithms are highly complex, especially when dealing with multi-omics data; more efficient AI algorithms are needed to handle the depth and complexity of this data. Insufficient interdisciplinary cooperation also poses a challenge, as the development of biomaterials involves multiple fields, including tissue engineering, 3D printing, genomics, and electronic information. There is an urgent need to enhance collaboration among these various disciplines. In terms of ethics and supervision, patient privacy protection and data security have become core issues. Meanwhile, the production and marketing of new biomaterials require strict approval processes, and relevant standards and norms still need further improvement.

Future research needs to focus on optimizing AI algorithms and material design to achieve more precise neural repair strategies. In material design, it is imperative to reference the latest findings in the field of nerve regeneration, such as the regulation of ferroptosis and pyroptosis (Chen et al., 2024a, b). Keeping pace with these cutting-edge achievements can provide a more substantial theoretical basis for the development of new types of biomaterials. Additionally, the application of AI in the medical field requires the support of big data. By revealing the insights and patterns identified in larger and more structured datasets, AI can provide valuable information for medical decision-making, therapy research, and the innovation of treatment methods. Therefore, enriching the clinical database and conducting big data analysis can reduce human bias and improve treatment accuracy. It also supports the precise regulation of material properties based on individual patient characteristics, facilitating the development of new treatment strategies and methods, and thereby achieving personalized treatment (Wan et al., 2023). By enhancing the capabilities of AI in virtual experiments and performance prediction, we can ensure that the repair materials precisely meet clinical needs. With the continuous development and deep integration of AI and materials science, the research, development, and clinical application of PNR materials are expected to witness significant growth, thereby accelerating the progress of neural regenerative medicine. By utilizing DL algorithms to analyze medical images and biological data, we can provide a more accurate diagnostic and therapeutic basis for neural regenerative medicine. Meanwhile, experts and scholars in related fields need to enhance interdisciplinary cooperation to develop PNR materials that can efficiently repair complex nerve injuries, significantly.

## Additional file:

***Additional Table 1:*** *Literature retrieval strategy.*

## Data Availability

*All relevant data are within the paper and its Additional files.*
